# Impact of COVID-19 Pandemic on Pre-Treatment Delays, Detection, and Clinical Characteristics of Tuberculosis Patients in Ningxia Hui Autonomous Region, China

**DOI:** 10.3389/fpubh.2021.644536

**Published:** 2021-05-21

**Authors:** Xiaolin Wang, Wencong He, Juan Lei, Guangtian Liu, Fei Huang, Yanlin Zhao

**Affiliations:** ^1^The Fourth People's Hospital of Ning Xia Hui Autonomous Region, Yinchuan, China; ^2^National Institute for Communicable Disease Control and Prevention, Chinese Center for Disease Control and Prevention, Beijing, China; ^3^National Center for TB Control and Prevention, Chinese Center for Disease Control and Prevention, Beijing, China

**Keywords:** COVID-19, tuberculosis, impact, TB control, pre-treatment delay

## Abstract

**Background:** To contain the pandemic of COVID-19, China has implemented a series of public health interventions that impacted the tuberculosis control substantially, but these impacts may vary greatly depending on the severity of the local COVID-19 epidemic. The impact of COVID-19 on TB control in Ningxia Hui Autonomous Region is little known.

**Methods:** Based on the national TB Information Management System (TBIMS), this study accessed the actual impact of COVID-19 on TB by comparing TB notifications, pre-treatment delays, and clinical characteristics of TB cases between 2020 COVID-19 period and 2017–2019 baseline. The data were divided into three periods based on the response started to fight against COVID-19 in Ningxia Hui Autonomous Region, including the control period (10 weeks before the pandemic), intensive period (10 weeks during the Ningxia Hui Autonomous Region lockdown), and regular (10 additional weeks after Ningxia Hui Autonomous Region reopen).

**Results:** TB notification dropped sharply in the first week of the intensive period but took significantly longer to return to the previous level in 2020 compared with the 2017–2019 baseline. Totally, the TB notification rates decreased by more than 60% in the intensive period of COVID-19 compared with the average level of 2017–2019. The sputum smear-positive rate of TB patients diagnosed in intensive period of COVID-19 was significantly higher than that in the corresponding periods of 2017–2019 (*P* < 0.001). The rate of cavity on X-ray inspection of TB cases diagnosed in the intensive period of COVID-19 was significantly higher than that in period 2 of 2017–2019 (23.5 vs. 15.4%, *P* = 0.004). The patients' delay in the intensive period was significantly longer than that before the pandemic (*P* = 0.047).

**Conclusions:** The TB notification in Ningxia was impacted dramatically by the pandemic of COVID-19. To compensate for the large numbers of missed diagnosis as well as delayed diagnosis during the intensive period of COVID-19, an urgent restoration of normal TB services, and further emphasis on enhanced active case finding and scale-up of household contact tracing and screening for TB-related symptoms or manifestation, will be essential.

## Introduction

Tuberculosis (TB) remains the leading killer from a single infectious organism worldwide and is responsible for 1.4 million deaths in 2019 (1). This severe situation may be exacerbated dramatically by the COVID-19 pandemic and related responses (2). A modeling study provided a stark warning that a decline in TB case detection caused by COVID-19 in 2020 could lead to a significant resurgence in TB deaths and brings us back to the level of TB mortality in 2015 (3).

In China, a series of public health interventions, mainly including the cancellation of all public transportation, the requirements for all residents to stay at home, the prohibition of public gathering, and the reassignments of TB-designated hospitals and personnel to receive COVID-19 patients, have successfully brought COVID-19 under control (4). However, these measures resulted in disruption of TB services, as well as restricting patients from seeking medical care, which have raised the public's concern, and this needs to be addressed (2, 5, 6). Substantial studies have reported the negative impacts of COVID-19 on TB control such as decline in TB detection, interruption in TB therapy, and decrease in TB treatment success ^1^, but most of them were from a national level or based on modeling predictions without real data as input (2, 7–9). There is almost no study fully discussing the impact of COVID-19 on TB control programs in a specific province, even though these impacts may vary greatly depending on the severity of local COVID-19 epidemic (10, 11).

To fill this knowledge gap, we investigated several indicators related to TB control in Ningxia Hui Autonomous Region. We compared the clinical characteristics of TB patients, TB notifications, patients delay, health system delay, and treatment delay from 2017 to 2020 before and after the pandemic of COVID-19 in China.

## Methods

### Settings

**Figure 1:**
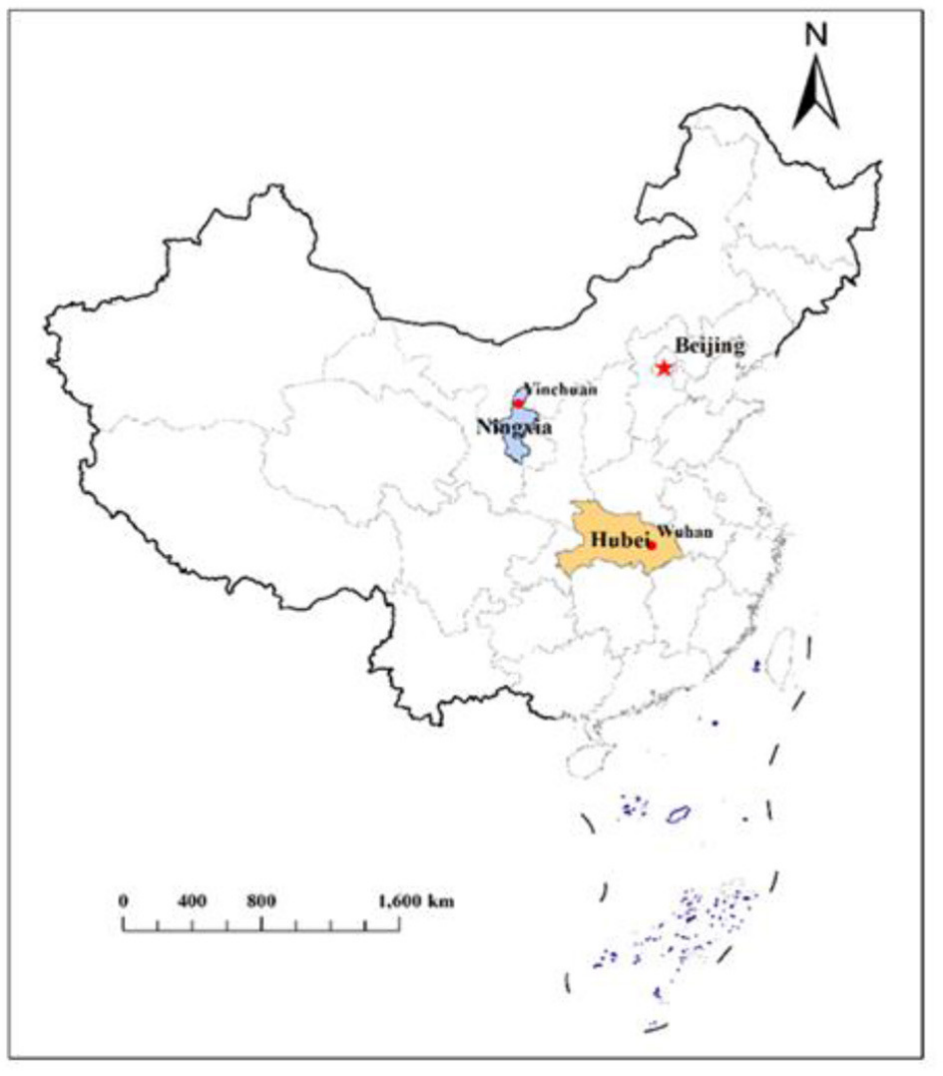
The geographical location of Ningxia Hui Autonomous Region in China.

The Ningxia Hui Autonomous Region is located in the northwest of China with a total area of more than 66,000 square kilometers (Figure 1). By the end of 2019, the resident population was 6,946,600, and the reported incidence of tuberculosis in 2020 was about 36 cases per 100,000 population^2^. Ningxia applied a mixed TB service model. At the county and prefecture levels, designated hospitals were developed to provide outpatient services for TB patients, but at the provincial level, there was a specialized TB hospital providing both clinical and public health services (12). On January 22, 2020, the first case of COVID-19 was confirmed in Ningxia Autonomous Region, which was from Wuhan, Hubei province (13). To lower the risk of further transmission, the government launched an urgent public health response and implemented a series of interventions from that day on, such as canceling public transportation, closing public spaces, requiring all residents to stay at home. As of March 1, 2020, a total of 75 confirmed cases and 20 suspected cases of COVID-19 were found (13). On April 1, 2020 (14 days after the discharge of the last COVID-19 case on March 16, 2020), people in Ningxia Hui Autonomous Region began to resume work and return to normal life, and hospitals returned to routine work gradually (13).

### Data Collection

In China, tuberculosis is a reportable communicable disease, and all TB patients are registered and managed through the national Tuberculosis Information Management System (TBIMS), which is an Internet-based and case-based information system, and the medical records of all TB patients are electronically stored in TBIMS. The medical records of TB patients from 2017 to 2020 were extracted from TBIMS on February 26, 2021, including clinical characteristics (e.g., treatment history, sputum smear results, X-ray inspection, etc.), follow-up examinations (e.g., sputum conversion after 2 months of treatment, treatment outcome, etc.), and medical history (e.g., date of symptoms onset, date of first seeking medical care, date of diagnosis, and date of initiating therapy, etc.).

### Key Time Points and Periods

**Figure 2:**
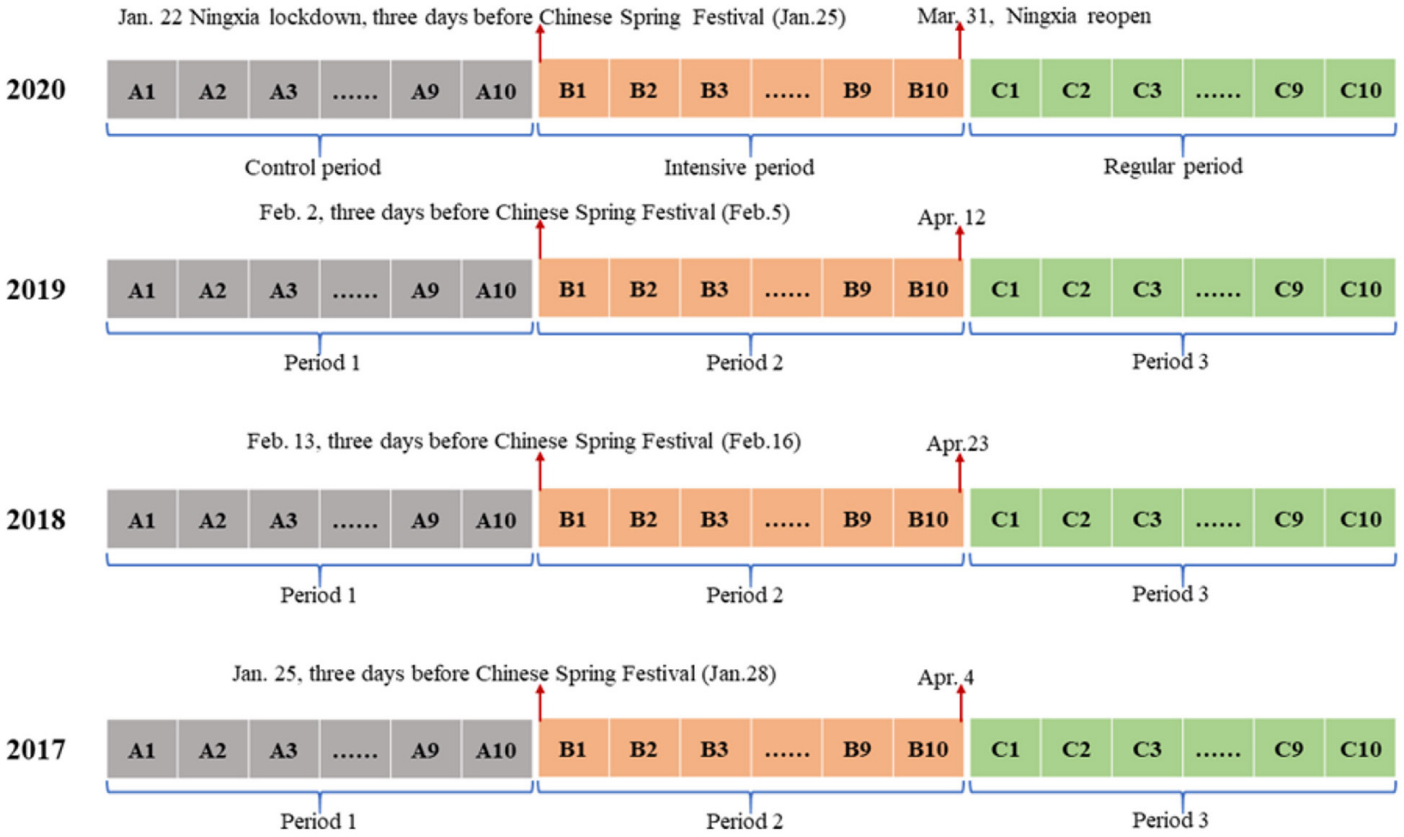
Key time points of three periods based on responses related to COVID-19 in the Ningxia Hui Autonomous Region.

To better understand the impact of COVID-19 on TB control in Ningxia Hui Autonomous Region, China, this study used three time periods based on two important time points: January 22, 2020 (the date when government began a stringent lockdown of Ningxia Hui Autonomous Region) and April 1, 2020 (Ningxia Hui Autonomous Region reopened). The period from January 22 to April 1, 2020 (10 weeks, B1–B10) was then considered as the intensive phase due to the COVID-19 epidemic. Ten weeks before January 22, 2020 (A1–A10) was considered as the control period without any interventions for COVID-19. The following 10 weeks after April 1 (C1-C10) was considered as the regular period of the epidemic. The 3 years prior to the epidemic, 2017–2019, were used as the baseline and were split into three counterpart periods (Period 1 to Period 3) by date (Figure 2).

In China, the Spring Festival lasting for 7 days is an important holiday. During this 1-week national vacation, most people avoid seeking medical care and instead reunite with family. Yet in 2020, after the first case of COVID-19 was confirmed in Ningxia Hui Autonomous Region on January 22 (3 days before Chinese Spring Festival), the local government decided to block this region to lower the risk for further transmission, indicating that this region has entered an intensive period of fighting COVID-19. Therefore, we selected the time point of 3 days before Chinese Spring Festival of 2017–2019 and 10 weeks before and 20 weeks after the time point as baseline.

### Definitions

Favorable outcomes were defined as cure or treatment completion, while other treatment outcomes such as failure, relapse, loss to follow-up, death, conversion to drug-resistance, and incompletion of treatment were defined as unfavorable outcomes.

Patient delay was defined as the time interval between the onset of TB-related symptoms and the patient's first visit to a healthcare provider.

Health system delay was defined as the time interval between the patient's first visit to a healthcare provider and the date of diagnosis.

Treatment delay was defined as the time interval between the date of confirmed diagnosis and the date of initiating therapy.

### Statistical Analysis

TB notifications, patient delay, health system delay, treatment delay, and clinical characteristics of TB cases were compared between 2017–2019 and 2020 broken down into three periods to reflect any changes due to the COVID-19 outbreak. The absolute number of weekly TB notifications was compared between 2020 and 2017–2019 baseline. TB notification rates per 100,000 of 2017–2019 and 2020 stratified by three periods were calculated and compared by using the TB notifications of the corresponding period as numerators and the beginning of the year populations as denominators. Chi-square test was used for categorical data, and Mann–Whitney *U*-test was used for continual data. All statistical analyses were performed in the SPSS version 18.0 software (SPSS Inc., Chicago, Illinois.) and R studio (version 3.6.1). All statistical tests were two-tailed, and *P* < 0·05 was considered statistically significant.

## Results

### Comparison of Clinical Characteristics of TB Cases by Three Periods Between 2020 COVID-19 Periods and 2017–2019 Baseline

During the three periods from 2017 to 2020, a total of 5,470 persons diagnosed with TB were notified and enrolled into the analysis in this study. The number of TB cases reported in the control, intensive, and regular periods of 2020 from TBIMS were 457, 197, and 419, respectively (Table 1). Clinical characteristics of patients such as treatment history, sputum conversion after 2 months, and treatment outcome varied a little between 2017–2019 and 2020 in period 1 (all *P* > 0.05), as well as in period 2 and period 3. In 2020, the sputum smear-positive rate of TB cases diagnosed in the control, intensive, and regular periods were 45.1, 54.6, and 48.2%, respectively, which was significantly higher than that in the corresponding periods of 2017–2019 (all *P* < 0.001). The rate of cavity on X-ray inspection of TB cases diagnosed in the intensive period of 2020 was significantly higher than that in period 2 of 2017–2019 (23.5 vs. 15.4%, *P* = 0.004), while in period 1 and period 3, no difference was observed in the cavity rate of TB cases between 2020 and 2017–2019 (15.8% vs. 13.0, *P* = 0.137; 12.2 vs. 13.9%, *P* = 0.389) (Table 1).

**Table 1:** Comparison of clinical characteristics of TB cases by three periods between the 2020 COVID-19 periods and 2017–2019 baseline.

**Variables**	**Period 1**			**Period 2**			**Period 3**		
	**2017–2019**	**2020**	***P***	**2017–2019**	**2020**	***P***	**2017–2019**	**2020**	***P***
	**(*N* = 1368)**	**(*N* = 457)**		**(*N* = 1,497)**	**(*N* = 197)**		**(*N* = 1,532)**	**(*N* = 419)**	
Treatment history			0.352			0.427			0.870
New cases	1,305 (95.4)	431 (94.3)		1,399 (93.5)	187 (94.9)		1,441 (94.1)	395 (94.3)	
Retreated cases	63 (4.6)	26 (5.7)		98 (6.5)	10 (5.1)		91 (5.9)	24 (5.7)	
Sputum smear results			<0.001			<0.001			<0.001
Positive	481 (35.2)	204 (45.1)		547 (36.5)	107 (54.6)		569 (37.3)	199 (48.2)	
Negative	886 (64.8)	248 (54.9)		950 (63.5)	89 (45.4)		958 (62.8)	214 (51.8)	
X-ray inspection			0.137			0.004			0.389
With cavity	176 (13.0)	72 (15.8)		230 (15.4)	46 (23.5)		212 (13.9)	51 (12.2)	
Without cavity	1,176 (87.0)	384 (84.2)		1,262 (84.6)	150 (76.5)		1,318 (86.1)	366 (87.8)	
Sputum conversion of 2 months treatment			0.593			0.230			0.198
Yes	400 (92.2)	170 (93.4)		456 (92.5)	88 (88.9)		470 (91.1)	162 (94.2)	
No	34 (7.8)	12 (6.6)		37 (7.5)	11 (11.1)		46 (8.9)	10 (5.8)	
Treatment outcomes^a^			0.078			0.560			0.058
Favorable outcomes	1,237 (90.4)	400 (87.5)		1,356 (90.6)	166 (89.2)		1,397 (91.2)	305 (87.9)	
Unfavorable outcomes	131 (9.6)	57 (12.5)		141 (9.4)	20 (10.8)		135 (8.8)	42 (12.1)	

a *Eleven and 72 TB cases diagnosed in period 2 and period 3 of 2020 were still on TB treatment, and these cases were excluded in the calculation and comparison of treatment outcomes in this study*.

### Comparison of Tuberculosis Notification Between 2020 COVID-19 Periods and 2017–2019 Baseline

**Figure 3:**
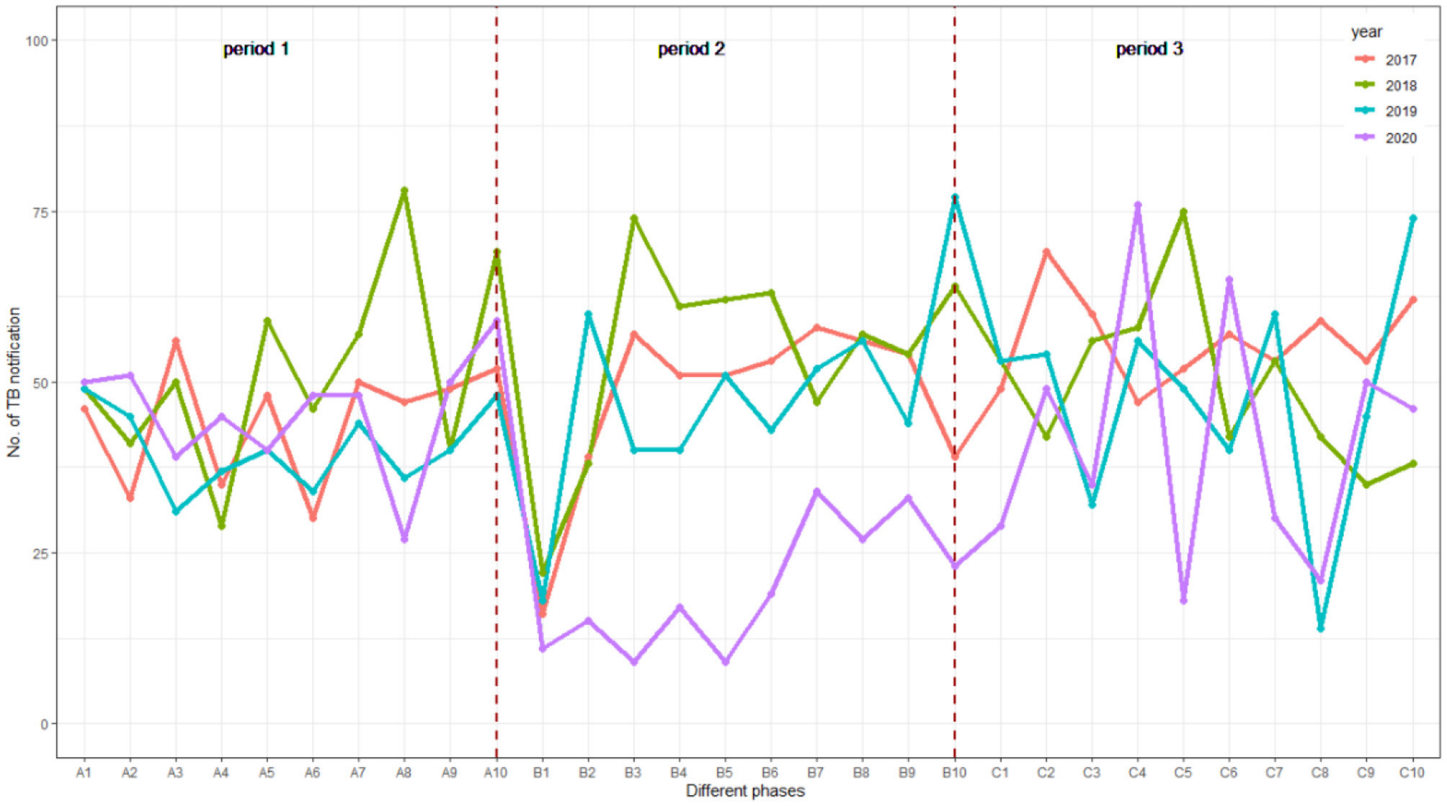
Weekly notification number for TB in 10 weeks before and 20 weeks after the Chinese Spring Festival, 2017–2020 in Ningxia, China.

The absolute number of weekly TB notifications in three periods from 2017 to 2020 is shown in Figure 3. Despite the fluctuations of weekly TB notifications in period 1, TB notifications in 2020 were consistent with the level of 2017–2019. The number in the first week of period 2 in 2017–2020 decreased sharply. However, the number of bounced back to previous level immediately in 2017–2019 increased gradually in 2020. In period 3, weekly TB notifications of the four tangled together as period 1 (Figure 3).

### Comparison of Tuberculosis Notification Rate Breakdown by Three Periods Between 2020 and 2017–2019 Average Level

The overall TB notification rate in three periods of 2020 decreased by more than 28% compared with the average level of 2017–2019. In period 1 before the epidemic of COVID-19, TB notification rate of 2020 was 6.6 per 100,000 population, similar to the 2017–2019 level (6.7 per 100,000 population). The TB notification rate of period 2 and period 3 in 2020 decreased by more than 60 and 19%, respectively, compared with that in the same period of 2017–2019 (Table 2).

**Table 2:** Comparison of TB notification rate breakdown by three periods between 2020 and 2017–2019 average level.

**Period**	**TB notification rate (95% CI)** ^**a**^		**Rate change ^**b**^**
	**2017–2019 average level [p1]**	**2020 [p2]**	
Period 1	6.7 (6.1-7.3)	6.6 (6.0-7.2)	−1.7%
Period 2	7.3 (6.7-8.0)	2.8 (2.4-3.2)	−61.3%
Period 3	7.5 (6.8-8.1)	6.0 (5.5-6.6)	−19.5%
Total	7.2 (6.8-7.5)	5.1 (4.8-5.5)	−28.2%

a *Rate per 100,000 population*;

b rate change = (p2 – p1)/p1^*^100%; CI, confidence interval.

### Comparison of Patients' Delay, Health System Delay, and Treatment Delay Breakdown by Three Periods Between 2020 and 2017–2019 Baseline

In period 1 and period 3, there was no significant difference in the distribution of patients delay between 2017–2019 and 2020, with *P*-value of 0.393 and 0.169, respectively. The median (interquartile, IQR) of patients delay during the intensive period of COVID-19 in 2020 were 29, which was significantly longer than the average patients delay in period 2 of 2017–2019. The distribution of health system delay in 2020 varied a little in all three periods compared with the average level of 2017–2019 in the corresponding period (all *P* > 0.05). The distribution of health treatment delay between 2017–2019 and 2020 was very similar; the median (IQR) of treatment delay in three periods of 2017–2019 and 2020 were all 0 (0–0) days (Table 3).

**Table 3:** Comparison of patient delay, health system delay, and treatment delay breakdown by three periods between 2017–2019 and 2020.

**Periods**	**Patient delay (days) [median (IQR)]**		***P***	**Health system delay (days) [median (IQR)]**		***P***	**Treatment delay (days) [median (IQR)]**		***P***
	**2017–2019**	**2020**		**2017–2019**	**2020**		**2017–2019**	**2020**	
Period 1	21 (9–42)	20 (8–50)	0.393	1 (0–6)	1 (0–6)	0.147	0 (0–0)	0 (0–0)	0.914
Period 2	23 (10–45)	29 (9–52)	0.047	1 (0–6)	1 (0–7)	0.399	0 (0–0)	0 (0–0)	0.932
Period 3	22 (9–44)	26 (8–49)	0.169	2 (0–7)	2 (0–6)	0.352	0 (0–0)	0 (0–0)	0.513

*IQR, interquartile*.

## Discussion

Our study assessed the actual impact of COVID-19 on TB by comparing TB notifications, pre-treatment delays, and clinical characteristics of TB cases between 2020 COVID-19 period and 2017–2019 baseline. We found that the TB notification rate in the intensive period of 2020 dropped by more than 60% compared with the same period of the previous 3 years. The percentage of TB cases with sputum smear positive and cavity on X-ray increased significantly in the intensive period of COVID-19, suggesting that the condition of these patients might be more severe. We also found that patients' delay during the intensive period of COVID-19 was significantly longer than that of 2017–2019.

In our study, before the pandemic of COVID-19, the number of weekly TB notifications in 2020 was basically the same as that of the baseline level, although the number of weekly TB notifications fluctuated greatly over time due to the small sample size. The weekly notifications of TB showed a sharp decline at the end of the Chinese Spring Festival holiday both in 2017–2019 and 2020, but rebounded soon afterward only in 2017–2019, which could explain the seasonality of TB with a prominent peak in spring reported by previous studies (14, 15). Seeking medical behavior is influenced significantly by the weeklong Spring Festival holiday, since most people, including TB suspected cases would stay at home to reunite with families rather than go to the hospital or clinics. In addition, many outpatient clinics of TB-designated hospitals were closed during this period. However, during the intensive period of COVID-19, the speed of weekly TB notifications rebounding to the baseline level was much slower than that in 2017–2019. This change was consistent with Huang's study based on the national surveillance data in China (16). There was a concern that, due to the disruption of health care and fear of contracting COVID-19, presumptive TB cases will avoid seeking health care when needed (10). With the mitigation of COVID-19, the TB notifications increased gradually and returned to the previous level, which suggested that the decrease in TB notification is temporary.

Substantial reduction in TB notifications caused by the pandemic of COVID-19 has been reported worldwide (16–19). China, as a country that initiated a series of public health interventions to contain the pandemic is no exception (7). In the present study, TB notification rates during the intensive period of COVID-19 decreased by more than 60% compared with the average level of 2017–2019, which was consist with other studies based on provincial surveillance data in China (10, 11). However, the lowered TB notification rate was not a reflection of actual reductions in TB incidences. To some extent, responses to COVID-19 adopted in Ningxia Hui Autonomous Region such as stringent quarantine and mask wearing might lower TB transmission levels in communities, which could lead to the decline in TB notification. However, this effect is unlikely to be observed immediately given the long incubation period of TB (20). Thus, the substantial drop in TB notification observed in this study could be explained by the following three reasons (18). First, TB service disruptions—some TB designated hospitals were closed and repurposed to fight against COVID-19 in Ningxia Hui Autonomous Region. Second, lack of access—during the lockdown period, most of the presumptive TB patients were prevented by movement restrictions from seeking medical services. Third, fear of COVID-19 infection—suspected TB cases might be reluctant to seek medical care for fear and stigma caused by the COVID-19 pandemic (4). These missed diagnosed TB patients would mean increased opportunities for further transmission, while the lack of appropriate treatment may worsen the clinical outcomes and increase the risk of death from TB (21).

Finding *Mycobacterium tuberculosis* in patients' specimens remains the gold standard for TB diagnosis (22). The “13th Five-Year Plan” of the National TB control Program issued by the Chinese government requires strengthening laboratory construction to increase the percentage of bacteriologically confirmed TB cases (16). Our study found that the rate of sputum smear positive in 2020 was significantly higher than the average level of 2017–2019, indicating the progress achieved in the TB program in China. Interestingly, we found that the sputum smear-positive rate during the intensive period of COVID-19 exceeded 54%, but decreased to 48% in the regular period of COVID-19. There are two possible explanations for the increase in smear-positive TB during the intensive period of COVID-19. One is that the improvement and strengthening of grassroots public health services during the intensive period of COVID-19 enables more sputum smear-positive cases to be identified in advance. Another is that during the intensive period of COVID-19, only these patients with severe symptoms will have to seek medical care, and they are more likely to be smear positive. In addition, we also found that the percentage of TB patients with cavity on X-ray increased significantly during the intensive period of COVID-19. Previous studies have suggested that TB patients with cavity have higher bacterial burdens and are more likely to release *M. tuberculosis* (23). This result further demonstrated the severer condition of TB patients diagnosed in the intensive period of COVID-19.

If not totally missed, delayed identification and diagnosis of TB cases also plays an important role in the transmission and aggravation of TB (24). The delay may be due to patients' delay if the patients could not visit a health facility timely after the onset of symptoms or health system delay if the patients are not diagnosed timely at the time of the first visit (25). The containment measures implemented in the whole country have induced disruption in TB services, and the restriction of traffic is impairing access to seeking medical care during the lockdown period of 2020 (26). Compared with the pre-lockdown period of COVID-19, the time of patients' delay in the intensive period of COVID-19 was significantly prolonged, which was consistent with the study of Huang et al. (16). As some symptoms of TB-suspected cases, such as cough and fever, are similar with COVID-19, suspected TB cases will be diagnosed with priority to exclude COVID-19 infection, which could explain why there was no significant delay in the health system during the intensive period of COVID-19. Moreover, in China, all TB patients diagnosed in designated hospitals will receive free anti-TB drugs and that is why there was no difference in days of treatment delay before and after the pandemic of COVID-19 (16).

One study from Italy suggested that the COVID-19 pandemic could worsen TB treatment outcomes significantly (27). However, in our study, no significant difference was observed in the treatment outcome between cohort patients diagnosed during the epidemic of COVID-19 and patients diagnosed in the 2017–2019 baseline. The larger number of patients still on treatment who were not included into comparison might confound this result. More importantly, during the intensive period of COVID-19, health care workers in Ningxia Hui Autonomous Region tried their best to ensure the continuous delivery of anti-TB drugs to TB patients, which helped patients to achieve favorable treatment outcomes.

The containment measures introduced to combat COVID-19 like social distancing and requirements for residents to stay indoors could reduce the transmission of TB in the community, but accelerate its transmission among household contacts (2). It is particularly alarming that TB patients, especially those with sputum smear positive and cavity on X-ray, are most likely to increase the risk of transmission within household members, medicated by delayed TB diagnosis and heavier exposure to TB source cases during household quarantine (5). More importantly, given that the previous studies showed that household contact with diabetes or aged <5 years old are more likely to develop active TB, preventive therapy in this vulnerable population should be taken into consideration (28, 29).

Our study has several limitations. First, the study was done in Ningxia, China. Variations in the background levels of TB and severity of local COVID-19 epidemic make our findings difficult to extrapolate to other areas or countries. Second, this study only focused on the short-term impact of COVID-19 pandemic on TB control, although McQuaid's study showed that the COVID-19 outbreak would affect TB control both in the short and long term (30). Third, some indicators related to severe TB were not included in this study, which prevent us from accessing the impact of COVID-19 on TB death systematically. Fourth, given that the situation of drug-resistant TB is not severe in this area, we did not evaluate the impact of COVID-19 on drug-resistant TB diagnosis and control.

Despite these limitations, our analysis suggests that the adverse impact of COVID-19 on TB control is substantial in Ningxia Hui Autonomous Region, China. Multiple-facet measures should be taken to mitigate the negative effects. On the one hand, in order to compensate for the large numbers of missed diagnosis and delayed diagnosis during the intensive period of COVID-19, an urgent restoration of normal TB services, and further emphasis on enhanced active case finding and scale-up of household contact tracing and screening for TB-related symptoms or manifestation, will be essential. On the other hand, online TB diagnosis and prescription of anti-TB drugs, with additional medicine delivery services should be widely introduced to confront the infectious disease like COVID-19 nowadays.

## Data Availability Statement

The data analyzed in this study is subject to the following licenses/restrictions: The datasets for this study are available from the corresponding author on reasonable request. Requests to access these datasets should be directed to zhaoyl@chinacdc.cn.

## Ethics Statement

The studies involving human participants were reviewed and approved by the Ethical Review Committee at the Fourth People's Hospital of Ning Xia Hui Autonomous Region, Yinchuan, China. Written informed consent to participate in this study was provided by the participants' legal guardian/next of kin.

## Author Contributions

XW, FH, and YZ conceptualized and designed this study. WH carried out the analysis and drafted the initial manuscript. JL and GL collected and organized the data. All authors were involved in revising the manuscript and approved the final manuscript as submitted and agreed to be responsible for all aspects of the work in ensuring that questions related to the accuracy or integrity of any part of the work are appropriately investigated and resolved.

## Conflict of Interest

The authors declare that the research was conducted in the absence of any commercial or financial relationships that could be construed as a potential conflict of interest.

## Footnotes

^1^https://theunion.org/news/the-union-shares-mid-term-report-on-impact-of-covid-19-on-people-with-tb-and-hivaids-in-africa.

^2^http://wsjkw.nx.gov.cn/zfxxgkzn/zdlygk/jbkz.htm.
